# Prevalence, awareness, treatment and control of hypertension, diabetes and hypercholesterolemia, and associated risk factors in the Czech Republic, Russia, Poland and Lithuania: a cross-sectional study

**DOI:** 10.1186/s12889-022-13260-3

**Published:** 2022-05-04

**Authors:** Wentian Lu, Hynek Pikhart, Abdonas Tamosiunas, Ruzena Kubinova, Nadezda Capkova, Sofia Malyutina, Andrzej Pająk, Martin Bobak

**Affiliations:** 1 Research Department of Epidemiology and Public Health, University College London, London, UK; 2 RECETOX, Masaryk University, Brno, Czech Republic; 3 Institute of Cardiology, Lithuanian University of Health Sciences, Kaunas, Lithuania; 4 National Institute of Public Health, Prague, Czech Republic; 5 Research Institute of Internal and Preventive Medicine, Branch of “Federal Research Center Institute of Cytology and Genetics”(IC&G), Siberian Branch of RAS, Novosibirsk, Russia; 6 Department of Epidemiology and Population Studies, Institute of Public Health, Faculty of Health Sciences, Jagiellonian University Medical College, Kraków, Poland

**Keywords:** Blood pressure, Fasting plasma glucose, Total cholesterol, Dyslipidemia, Central and Eastern Europe

## Abstract

**Background:**

Empirical evidence on the epidemiology of hypertension, diabetes and hypercholesterolemia is limited in many countries in Central and Eastern Europe. We aimed to estimate the prevalence, awareness, treatment and control of hypertension, diabetes and hypercholesterolemia in the Czech Republic, Russia, Poland and Lithuania, and to identify the risk factors for the three chronic conditions.

**Methods:**

We analysed cross-sectional data from the HAPIEE study, including adults aged 45–69 years in the Czech Republic, Russia, Poland and Lithuania, collected between 2002 and 2008 (total sample *N* = 30,882). Among prevalent cases, we estimated awareness, treatment, and control of hypertension, diabetes and hypercholesterolemia by gender and country. Multivariate logistic regression was applied to identify associated risk factors.

**Results:**

In each country among both men and women, we found high prevalence but low control of hypertension, diabetes, and hypercholesterolemia. Awareness rates of hypertension were the lowest in both men (61.40%) and women (69.21%) in the Czech Republic, while awareness rates of hypercholesterolemia were the highest in both men (46.51%) and women (51.20%) in Poland. Polish participants also had the highest rates of awareness (77.37% in men and 79.53% in women), treatment (71.99% in men and 74.87% in women) and control (30.98% in men and 38.08% in women) of diabetes. The common risk factors for the three chronic conditions were age, gender, education, obesity and alcohol consumption.

**Conclusions:**

Patterns of awareness, treatment and control rates of hypertension, diabetes and hypercholesterolemia differed by country. Efforts should be made in all four countries to control these conditions, including implementation of international guidelines in everyday practice to improve detection and effective management of these conditions.

**Supplementary Information:**

The online version contains supplementary material available at 10.1186/s12889-022-13260-3.

## Background

Hypertension, diabetes and hypercholesterolemia are important risk factors for cardiovascular diseases [1]. From 1980 to 2010, there were no major improvements in the prevalence of hypercholesterolemia and diabetes in Central and Eastern European (CEE) countries although the prevalence had been decreasing in Western Europe; CEE countries had the highest age-standardised mortality rates from cardiovascular disease, diabetes and chronic kidney disease attributable to the combined effects of high blood pressure, serum cholesterol, blood glucose and body mass index (BMI) in the world; and Russia kept ranking the third in contributing to these global attributable deaths in 1980–2010 [1]. Timely diagnosis, treatment and control of hypertension, diabetes and hypercholesterolemia in primary care is important in CEE countries.

Previous reports provided data on the prevalence, awareness, treatment and control of the three chronic conditions in CEE countries, but the majority of these studies focused on hypertension. For example, multiple investigations have been conducted in Poland and Russia among different age groups since the 1980s, consistently suggesting a high prevalence but low control rate of hypertension in both countries (e.g., prevalence of 67% versus 11% of control in Poland in 2007–2009; and prevalence of 87% versus 3% of control in Russia in 2015–2017) [2–9]. Studies in Poland also found a trend of increase in awareness, treatment and control rates of hypertension over time [2, 10], as well as lower prevalence of hypertension in seniors than the younger elderly [10, 11]. In the Czech Republic, two studies among adults aged 25–64 years since the 1980s indicated that the prevalence of hypertension had been decreasing among Czech women [12, 13], while another study reported a general decline in the prevalence of hypertension among both men and women over time [14]. All three Czech studies suggested increasing awareness, treatment and control rates of hypertension [12–14]. Findings on hypertension in Lithuania remain scarce. We found one study among urban Lithuanian adults aged 45–64 years in 1983–2009, reporting an increase and decrease in the prevalence of hypertension over time among men and women respectively, as well as increases in the awareness and treatment rates of hypertension [15].

Empirical evidence on diabetes and hypercholesterolemia in CEE countries is also limited. One Russian study among adults aged 40 + years in 2015–2017 found the prevalence of diabetes of 12%, similar to China and the United States, and high awareness of diabetes (73%) [16]. Another Russian study in men aged 25–60 years in 2008–2009 indicated a relatively low prevalence of hypercholesterolemia (45%) but less than 2% had been taking treatment [5]. Two Polish studies among adults aged 18 + years found a high prevalence of hypercholesterolemia (61% [17] and 70% in men and 64% in women [18], respectively), and low awareness, treatment and control rates of hypercholesterolemia [17].

In order to fill the evidence gap, we estimated the prevalence, awareness, treatment and control of hypertension, diabetes and hypercholesterolemia in the Czech Republic, Russia, Poland and Lithuania, using the baseline data of the Health, Alcohol and Psychosocial factors In Eastern Europe (HAPIEE) study [19]. The four CEE countries share similar history and societal context. Coinciding with the fall of communism, all four countries were experiencing political and economic transitions in the late 1990s and early 2000s. While the total and cardiovascular mortality have been declining in western Europe since the 1970s, in CEE and the former Soviet Union rates have been increasing, resulting in a large gap in life expectancy between eastern and western Europe [20]. Thus investigating these chronic conditions is essential for understanding more recent mortality patterns. Additionally, we also assessed the socio-demographic and behavioural risk factors for these chronic conditions.

## Methods

### Study populations and participants

We used data from the HAPIEE study [19] established in 2002–2005 to investigate the determinants of non-communicable diseases in urban populations in Russia (Novosibirsk), Poland (Krakow), six towns in the Czech Republic (Jihlava, Kromeriz, Liberec, Havirov/Karvina, Hradec Kralove and Usti nad Labem) and Lithuania (Kaunas, added in 2005–2008). The baseline survey used sampling frame at the individual level (i.e., a list of individuals, not households). The four HAPIEE cohorts consist of random samples of men and women aged 45–69 years at baseline, stratified by gender and 5-year age groups, and selected from the national population register in the Czech Republic, the city population registers in Krakow (Poland) and Kaunas (Lithuania), and the electoral list of two city districts of Novosibirsk (Russia). The overall response rate was 59%. HAPIEE recruited 8,857 Czech participants, 9,360 Russian participants, 10,728 Polish participants and 7,161 Lithuanian participants at baseline. The total sample size was 36,036 persons [19]. A total of 7,263 Czechs, 9,360 Russians, 9,285 Poles and 7,075 Lithuanians participated in the baseline medical examination.

Survey methods used in the four countries were summarised in Supplementary Table S1. Supplementary Figure S1 illustrates the procedure of sample selection. In the first step, we excluded the abnormal values of systolic blood pressure (SBP; < 70 or > 270 mmHg), diastolic blood pressure (DBP; < 30 or > 150 mmHg), fasting plasma glucose (FPG; < 2.5 or > 30 mmol/L) and total cholesterol (TC; < 1.75 or > 20 mmol/L). Blood samples were obtained from fasting participants. We also excluded participants who had incomplete data on mean SBP, mean DBP, FPG, TC, diagnosis and medication/treatment intake in each country. In the second step, we only included observations with complete data on all three chronic conditions to ensure comparability of results for the risk factors for each condition in the statistical analyses. The final analytical sample included 30,882 persons (6,130 Czechs, 9,137 Russians, 9,105 Poles and 6,510 Lithuanians).

### Definitions of prevalence, awareness, treatment and control

We employed the commonly used measures to define prevalent cases and prevalent cases with awareness, cases under treatment, and cases with their conditions controlled [21–23].

Hypertension was defined as having SBP of ≥ 140 or DBP of ≥ 90 mmHg or a self-report of taking antihypertensive medication. Awareness was defined as a self-report of having been diagnosed as having high blood pressure by a doctor. Treatment was defined as a self-report of taking antihypertensive medication. Control was defined as taking antihypertensive medication and having SBP of < 140 and DBP of < 90 mmHg.

Diabetes was defined as having FPG concentration of ≥ 7.0 mmol/L or a self-report of taking medication or insulin. Awareness was defined as a self-report of having been diagnosed as having diabetes by a doctor. Treatment was defined as a self-report of taking medication/insulin. Control was defined as having FPG concentration of < 7.0 mmol/L.

Hypercholesterolemia was defined as having TC of ≥ 5.0 mmol/L or a self-report of taking cholesterol-lowering medication. Awareness was defined as a self-report of having been diagnosed as having a high level of cholesterol by a doctor. Treatment was defined as a self-report of taking cholesterol-lowering medication. Control was defined as having TC of < 5.0 mmol/L. Specific lipid fractions were not considered in the current study. At the time of the baseline survey for HAPIEE, the general public were not knowledgeable about specific lipid fractions. For this reason, we asked only about the total cholesterol levels in the questions about whether having been diagnosed as having a high level of cholesterol by a doctor and having treatment for high cholesterol. These questions are essentially identical to those used in the MONICA project [24].

### Other covariates

Based on previous studies [7–9, 14, 16, 17, 21–23, 25–27], other covariates used in the analyses were age (in years), gender (men; women), marital status (married/cohabiting; single/divorced/separated; widowed), education (college education/above; secondary education; vocational education; primary education/below), the number of household amenities (low level: 0–3; middle level: 4–5; high level: 6–10), smoking (non-smokers; ex-smokers; current smokers), physical inactivity (self-reported vigorous activity < 2.5 h/week; no/yes), obesity (anthropometric measure; BMI ≥ 30 kg/m^2^; no/yes), and alcohol consumption (none; < 1/month; 1–3/month; 1–4/week; 5 + /week).

### Statistical analyses

We calculated the crude prevalence of hypertension, diabetes and hypercholesterolemia with 95% confidence interval (CI) among men and women separately in each country. Awareness, treatment and control rates with 95% CIs among prevalent cases, as well as treatment rates among cases with awareness, and control rates among cases under treatment, were also calculated with 95% CIs in men and women, separately. The prevalence was not standardised, since the age structure of the population samples in the four countries was virtually identical and the crude prevalence was directly comparable.

We also conducted multivariate logistic regression to identify factors associated with prevalence, awareness, treatment and control of the three chronic conditions. The independent variables for each regression model were age, gender, marital status, education, household amenities, smoking, physical inactivity, obesity, alcohol consumption and country (reference: the Czech Republic). Regression models for each outcome were fitted with adjustment for the remaining two outcomes’ prevalence as covariates. Odds ratio (OR) with 95% CI was calculated for each covariate. We also tested the statistical significance of differences in sample characteristics across countries.

Multiple imputation based on the chained equations was applied to handle missing values in the aforementioned covariates in each country [28]. All covariates and the variables for SBP, DBP, FPG, TC, diagnosis and medication/treatment intake were included in each imputation model for the prediction of missing values. Supplementary Figure S1 shows the number of missing values in each covariate. We generated fifty imputed datasets in each country but used imputed values of covariates only for regression modelling.

### Sensitivity analyses

To check the robustness of the main results, we re-calculated the epidemiological patterns of the three chronic conditions in each country based on the maximum number of participants (i.e., different sample sizes that we defined in the first step of data selection; supplementary Figure S1), which were larger than that in the main analyses. We compared the results of the main versus sensitivity analyses.

Our analyses were not performed with a complex design module. Weighting adjustment was not applied. Each HAPIEE cohort selected samples randomly within each age- and gender-stratified group. In each country, the sub-national population was not stratified by small geographical units and clusters (smaller areas in a region) within each stratum.

All analyses were performed using STATA MP 16.1 [29], with a P-value threshold of < 0.05 for statistical significance.

## Results

Table 1 presents sample characteristics by country. Russian participants had higher mean values of SBP, DBP, FPG and TC than other countries’ participants. The proportions of having self-reported diagnosed high blood pressure were higher in Russia (59.28%) and Lithuania (59.63%) than Poland (53.33%) and the Czech Republic (45.09%), whereas the proportion of antihypertensive medication intake was lower in Russia (54.12%) than other countries. Russia also had the lowest proportions of having self-reported diagnosed diabetes (5.28%) and high cholesterol (15.31%). However, the proportion of medication intake for lowering high cholesterol in Russia was also the lowest (23.80%) in the four countries. In terms of other variables, Lithuanian participants were less likely to be married/cohabiting, younger and not obese, but more likely to be highly educated, physically active, non-smokers and non-drinkers, and have a high level of household amenities than other countries’ participants. Russian participants had the largest proportion of having a low level of household amenities (51.58%). More than 30% of Polish participants were current smokers and more than 12% of Czech participants consumed 5 + times of alcohol drinks per week.

**Table 1:** Sample characteristics by country (unweighted)

Variable	Czech Republic (*N* = 6130)	Russia (*N* = 9137)	Poland (*N* = 9105)	Lithuania (*N* = 6510)
	**Mean (S.D.)**	**Mean (S.D.)**	**Mean (S.D.)**	**Mean (S.D.)**
**Age**	58 (7.10)	58 (7.09)	57 (6.98)	61 (7.54)
**Systolic blood pressure (mmHg)**	139.36 (19.71)	142.74 (24.58)	138.38 (21.29)	139.81 (21.73)
**Diastolic blood pressure (mmHg)**	88.85 (10.72)	89.97 (13.27)	86.33 (11.83)	89.54 (12.34)
**Fasting plasma glucose (mmol/L)**	5.93 (1.48)	6.01 (1.67)	5.38 (1.46)	5.83 (1.22)
**Total cholesterol (mmol/L)**	5.74 (1.05)	6.28 (1.27)	5.84 (1.10)	5.96 (1.14)
	**%**	**%**	**%**	**%**
**Gender**				
Men	45.66	45.51	48.68	44.64
Women	54.34	54.49	51.32	55.36
**Marital status**				
Married/Cohobating	76.53	72.30	76.93	69.49
Single/Divorced/Separated	14.56	14.15	12.72	16.37
Widowed	8.92	13.55	10.34	14.13
**Education**				
College education/above	13.79	28.81	29.04	55.88
Secondary education	36.91	34.20	38.83	24.58
Vocational education	37.15	26.53	20.80	7.07
Primary education/below	12.16	10.46	11.33	12.47
**Household amenities**				
High level	36.12	15.39	33.09	40.61
Middle level	39.84	33.03	40.82	38.60
Low level	24.04	51.58	26.09	20.79
**Smoking**				
Non-smokers	44.49	58.21	40.76	63.46
Ex-smokers	30.02	13.67	29.11	17.86
Current smokers	25.49	28.12	30.13	18.68
**Physical inactivity (vigorous activity < 2.5 h/week)**				
No	86.20	93.40	89.92	94.50
Yes	13.80	6.60	10.08	5.50
**Obesity**				
No	69.55	65.09	69.15	59.66
Yes	30.45	34.91	30.85	40.34
**Alcohol consumption**				
None	11.75	15.95	33.60	47.86
< 1/month	26.45	38.17	23.87	26.85
1–3/month	20.61	21.29	20.06	19.49
1–4/week	28.38	22.03	18.58	5.35
5 + /week	12.81	2.56	3.89	0.45
**Self-reported diagnosed high blood pressure**				
No	54.91	40.72	46.67	40.37
Yes	45.09	59.28	53.33	59.63
If yes, taking medication for high blood pressure	79.96	54.12	71.23	67.10
**Self-reported diagnosed diabetes**				
No	89.05	94.72	88.69	92.58
Yes	10.95	5.28	11.31	7.42
If yes, taking medication/insulin for diabetes	47.54	64.94	65.73	74.33
**Self-reported diagnosed high cholesterol**				
No	67.80	84.69	57.45	74.25
Yes	32.20	15.31	42.55	25.75
If yes, taking medication for high cholesterol	42.50	23.80	43.80	34.96

Table 2 and Table 3 show the prevalence, awareness, treatment and control of hypertension, diabetes and hypercholesterolemia by country and gender. For hypertension, in all countries, the prevalence was over 60% and 55% in men and women. Among these prevalent cases, Russian, Polish and Lithuanian men and women’s awareness rates were more than (or around) 70% and 80%, respectively; whereas in the Czech Republic, men’s and women’s awareness rates were less than (or around) 60% and 70%. More than (or around) 50% of male prevalent cases in the Czech Republic, Poland and Lithuania, and more than 60% of female prevalent cases in the four countries received treatment; whereas less than 35% of male prevalent cases in Russia received treatment. The control rates among these prevalent cases in men and women in all countries were less than (or around) 15% and 25%. More than 80% of Czech men and Czech, Polish and Lithuania women who were aware of having hypertension received treatment. Less than 30% and 40% of men and women who received treatment successfully controlled their hypertension in each country. Across countries, Russian men had a lower prevalence but also lower awareness, treatment and control rates than men in other countries. Among women, Russian participants had the highest prevalence but the lowest treatment and control rates, while Polish participants had the lowest prevalence but the highest treatment and control rates.

**Table 2:** Prevalence, awareness, treatment and control of hypertension, diabetes and hypercholesterolemia in men by country (unweighted)

	**Czech Republic**	**Russia**	**Poland**	**Lithuania**
	**Proportion (95%CI)**	**Proportion (95%CI)**	**Proportion (95%CI)**	**Proportion (95%CI)**
**Hypertension**	**%**	**%**	**%**	**%**
Prevalence	73.31 (71.64–74.92)	63.28 (61.80–64.73)	66.58 (65.18–67.96)	73.09 (71.45–74.67)
Awareness	61.40 (59.28–63.49)	68.34 (66.53–70.09)	72.38 (70.74–73.97)	75.75 (73.88–77.53)
Treatment	49.46 (47.30–51.63)	34.70 (32.90–36.54)	55.27 (53.47–57.06)	48.87 (46.75–51.00)
Treatment among awareness	80.56 (78.27–82.65)	50.78 (48.47–53.09)	76.36 (74.51–78.11)	64.51 (62.14–66.82)
Control	10.38 (9.13–11.78)	7.45 (6.51–8.52)	14.94 (13.70–16.28)	10.64 (9.40–12.03)
Control among treatment	20.99 (18.59–23.60)	21.47 (18.92–24.25)	27.04 (24.94–29.25)	21.77 (19.36–24.39)
**Diabetes**	**%**	**%**	**%**	**%**
Prevalence	16.68 (15.35–18.11)	10.70 (9.80–11.68)	12.16 (11.23–13.16)	10.56 (9.50–11.74)
Awareness	54.18 (49.62–58.66)	30.79 (26.66–35.24)	77.37 (73.63–80.71)	55.05 (49.42–60.55)
Treatment	41.97 (37.56–46.51)	24.49 (20.71–28.72)	71.99 (68.03–75.62)	49.84 (44.25–55.43)
Treatment among awareness	77.47 (71.88–82.22)	79.56 (71.91–85.55)	93.05 (90.16–95.13)	90.53 (85.06–94.14)
Control	11.13 (8.58–14.33)	4.72 (3.09–7.14)	30.98 (27.21–35.03)	15.64 (11.97–20.16)
Control among treatment	26.53 (20.79–33.19)	19.27 (12.85–27.86)	43.04 (38.18–48.04)	31.37 (24.47–39.21)
**Hypercholesterolemia**	**%**	**%**	**%**	**%**
Prevalence	76.67 (75.07–78.20)	80.47 (79.24–81.65)	80.10 (78.90–81.25)	77.67 (76.12–79.15)
Awareness	36.02 (34.01–38.08)	11.24 (10.21–12.35)	46.51 (44.87–48.15)	22.86 (21.18–24.64)
Treatment	17.19 (15.66–18.85)	3.89 (3.28–4.60)	20.73 (19.43–22.10)	9.57 (8.42–10.86)
Treatment among awareness	47.74 (44.23–51.27)	34.57 (29.92–39.54)	44.58 (42.19–46.99)	41.86 (37.66–46.18)
Control	5.27 (4.40–6.30)	0.57 (0.36–0.89)	5.13 (4.45–5.90)	2.17 (1.64–2.86)
Control among treatment	30.62 (26.12–35.53)	14.62 (9.48–21.86)	24.73 (21.74–27.98)	22.69 (17.56–28.78)

**Table 3:** Prevalence, awareness, treatment and control of hypertension, diabetes and hypercholesterolemia in women by country (unweighted)

	**Czech Republic**	**Russia**	**Poland**	**Lithuania**
	**Proportion (95%CI)**	**Proportion (95%CI)**	**Proportion (95%CI)**	**Proportion (95%CI)**
**Hypertension**	**%**	**%**	**%**	**%**
Prevalence	59.08 (57.40–60.74)	66.88 (65.56–68.18)	55.77 (54.34–57.19)	62.07 (60.47–63.64)
Awareness	69.21 (67.13–71.21)	85.71 (84.47–86.85)	81.93 (80.40–83.36)	86.23 (84.74–87.60)
Treatment	60.72 (58.54–62.86)	60.60 (58.93–62.25)	70.11 (68.32–71.84)	70.05 (68.12–71.91)
Treatment among awareness	87.74 (85.89–89.38)	70.71 (69.01–72.35)	85.57 (84.02–87.00)	81.23 (79.43–82.92)
Control	22.21 (20.42–24.10)	15.56 (14.36–16.83)	26.48 (24.82–28.21)	22.84 (21.15–24.63)
Control among treatment	36.57 (33.88–39.34)	25.67 (23.81–27.62)	37.77 (35.57–40.02)	32.61 (30.33–34.97)
**Diabetes**	**%**	**%**	**%**	**%**
Prevalence	9.46 (8.51–10.50)	10.58 (9.76–11.47)	8.26 (7.50–9.08)	11.15 (10.17–12.22)
Awareness	54.92 (49.37–60.35)	45.16 (40.95–49.45)	79.53 (75.20–83.28)	59.70 (54.81–64.40)
Treatment	39.05 (33.79–44.57)	38.71 (34.63–42.95)	74.87 (70.29–78.96)	51.24 (46.35–56.12)
Treatment among awareness	71.10 (63.86–77.40)	85.71 (80.64–89.63)	94.14 (90.87–96.28)	85.83 (80.80–89.72)
Control	6.35 (4.13–9.65)	8.16 (6.10–10.83)	38.08 (33.36–43.05)	16.17 (12.88–20.11)
Control among treatment	16.26 (10.69–23.95)	21.08 (15.99–27.25)	50.87 (45.09–56.62)	31.55 (25.54–38.25)
**Hypercholesterolemia**	**%**	**%**	**%**	**%**
Prevalence	82.89 (81.57–84.13)	89.60 (88.72–90.41)	85.77 (84.74–86.74)	86.18 (85.02–87.27)
Awareness	39.62 (37.81–41.46)	21.48 (20.29–22.70)	51.20 (49.65–52.74)	34.96 (33.31–36.66)
Treatment	17.02 (15.67–18.47)	4.55 (3.98–5.20)	23.98 (22.68–25.32)	11.91 (10.82–13.10)
Treatment among awareness	42.96 (40.05–45.92)	21.19 (18.71–23.90)	46.83 (44.68–49.00)	34.07 (31.31–36.95)
Control	4.17 (3.48–4.98)	0.36 (0.22–0.58)	3.97 (3.40–4.62)	1.48 (1.11–1.97)
Control among treatment	24.47 (20.78–28.57)	7.88 (4.87–12.51)	16.55 (14.32–19.03)	12.43 (9.43–16.22)

For diabetes, the prevalence was under 15%, except for Czech men, in whom the prevalence was around 17%. Among these prevalent cases, only Polish men and women had awareness and treatment rates larger than 70%. The control rates among Polish men and women were greater than 30% whereas the control rates among both men and women in other countries were less than 20%. Especially in Russian men and women, and Czech women, the control rates were less than 10%. Across all countries, more than 70% of men and women who were aware of having diabetes received treatment. For those who received treatment, less than (or around) 50% of Polish men and women, and less than (or around) 30% of men and women in other countries, successfully controlled their diabetes. Across countries, Polish participants had the highest rates of awareness, treatment and control than participants and Russian men had the lowest rates of awareness and treatment.

The prevalence of hypercholesterolemia was more than 75% and 80% in men and women. Among prevalent cases, only Polish women’s awareness rate was larger than 50%. The treatment and control rates among these prevalent cases in both men and women in all countries were less than 25% and 6%. Less than 50% of men and women who were aware of having hypercholesterolemia received treatment in each country. Less than (or around) 30% and 25% of men and women who received treatment successfully controlled their hypercholesterolemia in each country. In both men and women across countries, Russian and Lithuanian participants had lower rates of awareness, treatment and control than Czech and Polish participants. In Russia, the control rates among these prevalent cases were less than 1% in both men and women.

**Fig. 1:**
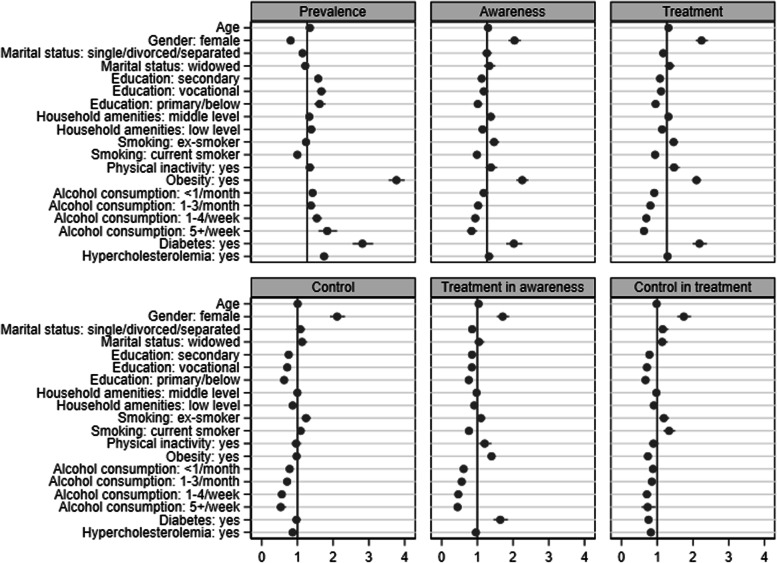
Odds ratio with 95% confidence interval for each risk factor associated with the epidemiology of hypertension in a combined sample

**Fig. 2:**
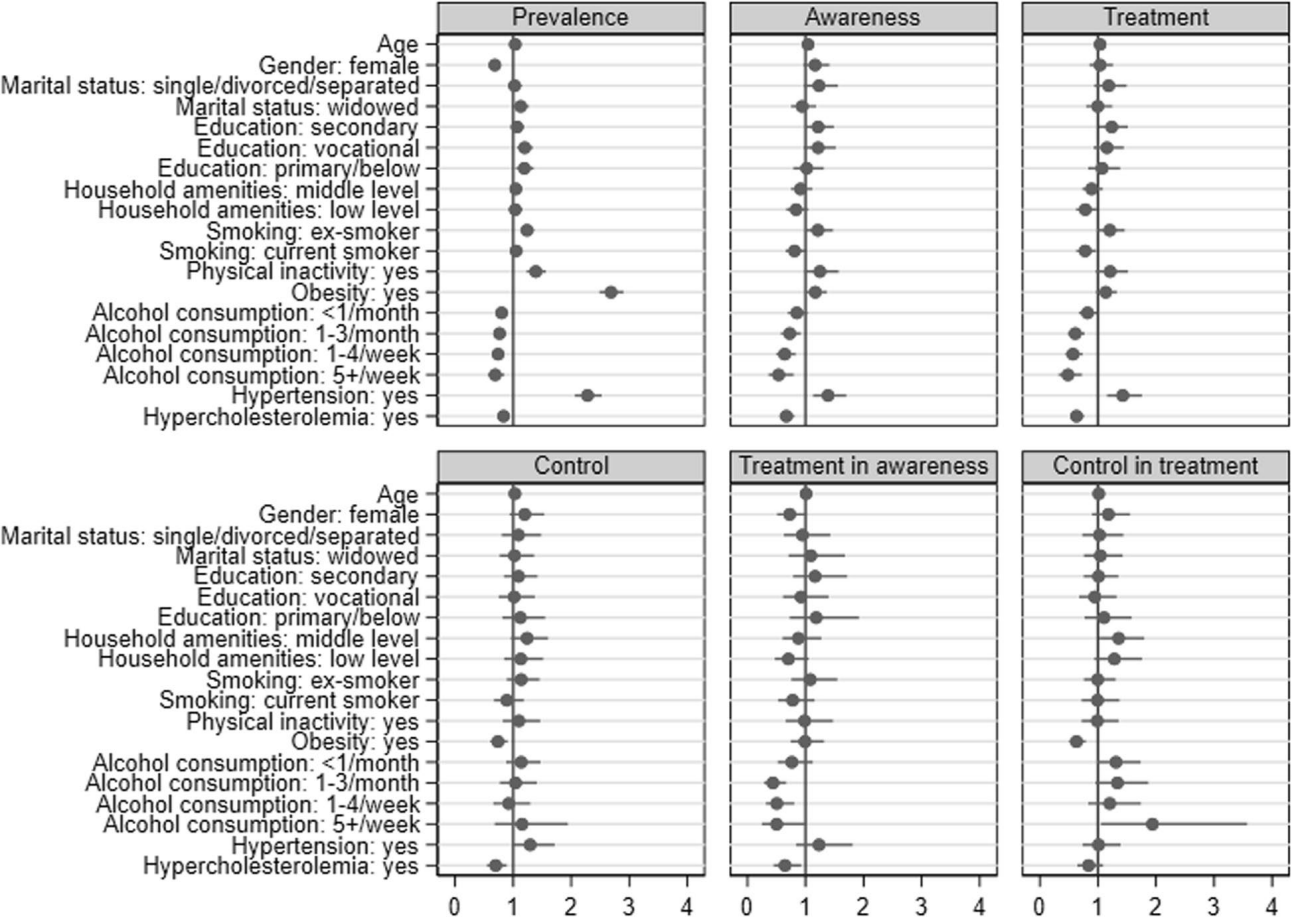
Odds ratio with 95% confidence interval for each risk factor associated with the epidemiology of diabetes in a combined sample

**Fig. 3:**
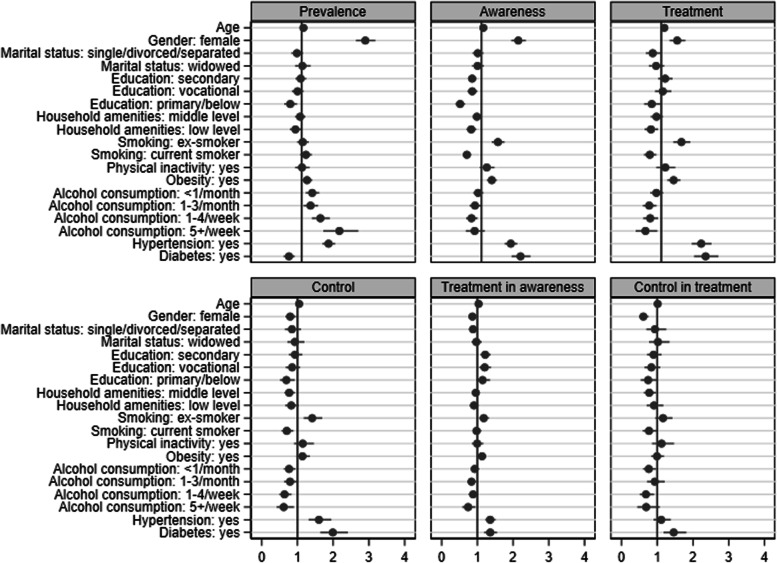
Odds ratio with 95% confidence interval for each risk factor associated with the epidemiology of hypercholesterolemia in a combined sample

Figure 1, Fig. 2 and Fig. 3 illustrate the associations (ORs with 95% CIs) of covariates with the indicators of hypertension, diabetes and hypercholesterolemia in the combined sample of four countries. Being older, male, married/cohabiting, low-educated, obese, non-smoker, alcohol consumer, and having a low level of household amenities, diabetes and hypercholesterolemia, was associated with higher odds of hypertension. Being older, male, widowed, low-educated, ex-smoker, physically inactive, obese, and non-alcohol consumer, and having hypertension but no hypercholesterolemia, was associated with higher odds of diabetes. Hypercholesterolemia was associated with older age, female sex, higher education, obesity, alcohol consumption, a higher level of household amenities, and hypertension (but no diabetes). Compared with Czech participants, Polish participants had a significantly lower prevalence of hypertension; and Russian, Polish and Lithuanian participants also had a significantly lower prevalence of diabetes but a higher prevalence of hypercholesterolemia.

Supplementary Figure S2 shows the differences in study sample characteristics across countries. In general, there were significant differences in mean values of age, SBP and DBP, and proportions of alcohol consumption and household amenities across countries.

Supplementary Table S2 and Table S3 show the results of sensitivity analyses. In each country, and among both men and women, estimations of prevalence, awareness, treatment and control of hypertension, diabetes and hypercholesterolemia in sensitivity analyses were very similar to those in the main analyses, suggesting that findings were robust to different inclusion criteria.

## Discussion

### Summary of key findings

We found that in all four countries, the prevalence rates of hypertension, diabetes and hypercholesterolemia were high while the control rates of the three chronic conditions were low. There were variations in the rates of awareness, treatment and control of the three chronic conditions between countries and between men and women. The most consistent correlates of all indicators for the three chronic conditions were age, gender, education, obesity and alcohol consumption.

### Results in the context of other studies

Our findings of the high prevalence but low control rates of hypertension in the four countries were consistent with previous reports from Poland and Russia [2–9]. We also found that Russian women had a higher prevalence of hypertension (67%) than women in other countries; and both Russian men and women had lower control rates (7% in men; 16% in women) than participants in other countries. This pattern seems to persist. In a recent cross-sectional study among Russian individuals aged 18 + years, compared with other countries worldwide, Russian participants still had a higher prevalence of hypertension, a comparable treatment rate, but a worse rate of control [4]. The global report in 2021 suggested that treatment and control rates improved most in high-income western and central European countries from 1990 to 2019. But the prevalence and control rates of hypertension in CEE countries remained relatively high and low respectively over years (e.g., prevalence in 2019: 77% in women and 63% in men; control rate in 2019: 25% in women and 17% in men) [30].

Our findings of the prevalence of diabetes in Russia (men and women: 11%) in 2002–2005 was similar to the findings of the Russian study in 2015–2017 among participants in a similar age range, where the prevalence of diabetes was 12% [16]. However, we found distinctly lower awareness rates (men: 31%; women: 45%) and control rates in those taking treatment (men: 19%; women: 21%) than that in the other Russian study (awareness: 73%; control among treatment: 59%) [16], which might be due to different time periods of the two studies. It seemed to indicate that the prevalence of diabetes did not change remarkably while the awareness and control rates of diabetes had been increasing over time among adults aged 40 + in Russia. We also found that Polish participants had higher rates of awareness, treatment and control than participants in other countries; and their awareness rates in 2002–2005 (men: 77%; women: 80%) were even higher than that in Russia in 2015–2017 [16].

Our findings of the high rates of prevalence but low rates of awareness, treatment and control of hypercholesterolemia among both men and women in the four countries were similar to the findings of the two earlier Polish studies [17, 18]. A Russian study among men aged 25–60 years in 2008–2009 reported a relatively low prevalence of hypercholesterolemia (45%) [5], whereas, in our study, the prevalence was 80% in Russian men aged 45–69 years in 2002–2005. However, we commonly found very low treatment rates in Russian men with hypercholesterolemia (< 2% in previous work [5] and 4% in our study). A global report in 2020 indicated that between 1980 and 2018, the age-standardised mean total cholesterol in both men and women in high-income western and CEE countries decreased most, suggesting that the rates of awareness, treatment and control of hypercholesterolemia might be increasing over time. However, further preventive and treatment efforts are still needed since the mean total cholesterol level in these countries was still distinctly higher than many countries in Asia, Africa and Latin America [31].

The four CEE cohorts included in our study had distinctly higher prevalence but lower control rates of hypertension than most high-income countries. A systematic review covering the period of 1980–2003 indicated that in high-income countries such as the United States, Canada, Germany, England, and Japan, the prevalence of hypertension ranged between 20 and 50% [32]. Another study among adults aged 40–79 years old in twelve high-income countries suggested that in early and mid-2000s, the control rates in Canada and Germany were between 50 and 58% in women and between 48–69% in men [33], which were substantially higher than that in our study. The prevalence rates of diabetes in our CEE cohorts were also higher than the worldwide average in 2014 (9% in men and 8% in women) [34]. Only in Poland the control rates of diabetes were over (or around) 30%, which was higher than the control rate of 26% in a South Korean study in 2013 [35]. The MONICA project in nineteen countries in 1989–1997 found that on average the prevalence rates of hypercholesterolemia among participants aged 35–64 years old were 27% in men and 25% in women [24]. The World Health Organization estimated the global prevalence of the raised total cholesterol (defined as TC ≥ 5.0 mmol/l) among adults aged 18 + as 39% (37% for men and 40% for women) in 2008 [36]. Although results across studies are not fully comparable due to different age distributions, the prevalence of over 75% in men and 80% in women for hypercholesterolemia in our study is high.

### Risk factors for hypertension, diabetes, and hypercholesterolemia

There were gender differences in the patterns of the three chronic conditions. Consistent with previous findings in the Czech Republic and Poland [9, 14, 25], we found the prevalence of hypertension was significantly lower, but the awareness, treatment and control rates were significantly higher in women than men. Two cross-country comparative studies also suggested that in both Latin American and high-income countries such as the US and England, women had higher awareness, treatment, and control rates of hypertension than men [32, 33]. We also found a significantly lower prevalence of diabetes in women than men, but no differences in awareness, treatment and control rates between men and women. While a study in Kazakhstan (2012–2015) suggested substantially higher rates of awareness, treatment and control of diabetes in women than men [22]. Differently, we found women had a significantly higher prevalence of hypercholesterolemia than men; although the awareness and treatment rates of hypercholesterolemia were higher in women, the control rates were significantly lower in women than men. These findings were consistent with findings of a study in Kazakhstan (2012–2015) [23]. While a study in Poland in 2011 reported no gender difference in the prevalence of hypercholesterolemia [17].

Apart from gender, age, education, obesity and alcohol consumption were also common risk factors for the epidemiology of the three chronic conditions. Consistently, previous studies suggested that having older age and obesity were associated with a higher prevalence of hypertension [7–9, 21], diabetes [16, 22, 26] and hypercholesterolemia [17]. We also found that being low-educated was associated with a higher prevalence of hypertension, and lower rates of awareness, treatment and control of hypertension and hypercholesterolemia than those being high-educated, which were consistent with previous findings [8, 9, 23]. However, in our study, individuals with disadvantaged socioeconomic position (being low-educated or having a low level of household amenities) were less likely to have hypercholesterolemia than those with advantaged socioeconomic position. A study in Brazil also showed a positive relationship between socioeconomic position and the risk of dyslipidemia [27]. In our study, we also found that alcohol consumers had a significantly higher prevalence of hypertension and hypercholesterolemia, but lower rates of awareness, treatment and control of hypertension, hypercholesterolemia and diabetes, than non-alcohol consumers. One study in Russia and another study in Argentina, Chile and Uruguay also indicated positive associations between alcohol consumption and the prevalence of hypertension [8, 37]. Alcohol consumption is an important risk factor for cardiovascular disease and total mortality in CEE countries. In 2003 in a typical Russian city, around half of all deaths in working-age men were attributed to hazardous drinking [38]. An association of binge drinking with high blood pressure was previously reported from the HAPIEE study [39]. National alcohol policies since 2004 in Russia, such as alcohol market restrictions, monitoring alcohol production, a ban on internet alcohol sales, or tax increase, has played a central part in the decrease of hypertension prevalence [38]. The prevalence of hypertension in Russia decreased to around 40% in men and 45% in women in 2019 [30].

### Limitations

This study has several limitations. Firstly, we relied on self-reported diagnosed high blood pressure, diabetes and high cholesterol, and medication/treatment intake to define awareness and treatment and to calculate control rates. Recall bias may exist [40].

Secondly, we excluded participants with abnormal or missing values for SBP, DBP, FPG, TC, diagnosis and medication/treatment intake in each country. We also only included observations with complete data on all three chronic conditions in the main analyses. The distributions of some covariates might be altered and the variations in risk factors might also be reduced. However, we found very little differences in the sample characteristics between observations who were included and excluded from the main analyses (Supplementary Table S4). Therefore, it is unlikely that the exclusion of abnormal or missing values and the sample restriction introduce a major selection bias. Our key findings were consistent both across the four countries and with the literature.

Thirdly, our study is cross-sectional. The issue of reverse causation could influence multiple key exposures in our study [41]. For example, we found current smokers and alcohol consumers were less likely to have hypertension and diabetes than non-smokers and non-alcohol consumers, respectively. Previous work also found that smoking was not associated with a high risk of hypertension [21, 26]. Individuals with hypertension or diabetes might be more willing to stop using tobacco and consuming alcohol, which potentially alters the strength and direction of the association between smoking/alcohol consumption and chronic conditions [41].

Fourthly, although the HAPIEE samples were randomly selected from the population registers, they are based on urban populations. These populations are therefore not nationally representative. Urban populations are more likely to have better access to high quality health care than rural population, leading to an overestimation of the rates of awareness, treatment and control. In addition, the overall response rate was 59% across the four study centres, and there was additional non-response to medical examination in Czech Republic and Poland. The literature consistently suggests that compared to non-respondents, respondents have better health status and more favourable risk profile in general [42]. It is very likely that this selection bias contributed an underestimation of the prevalence of risk factors. The situation in the whole countries may be less favourable than what we found in the current study.

Finally, our data was collected before 2010. Thus, the findings reflected the situation during that specific period. However, it is unlikely that the control rates of these conditions have improved dramatically since then. Additionally, to our best knowledge, there have been very limited evidence on the epidemiology of diabetes, hypercholesterolemia and, to a lesser extent, hypertension in the four CEE countries. Findings in our study filled this research gap. Understanding the epidemiological patterns of these chronic conditions in 2000s is important, as they affect the current prevalence rates of cardiovascular disease in CEE region.

### Implications

The substantially high prevalence of hypercholesterolemia in CEE countries highlighted the urgency of implementing effective interventions for the inadequate awareness, treatment and control of hypercholesterolemia. Although the treatment rates among those who were aware of having diabetes were high, more advanced and multifaceted interventions for diabetes management are still needed to increase the awareness and control rates in the four countries [43], especially in the Czech Republic, Russia and Lithuania. For hypertension care, a survey in 2012 revealed that physicians in CEE countries declared lower treatment goals, as well as the immediate initiation of pharmacotherapy at lower blood pressure level, than that was recommended in international guidelines [44]. The efforts to implement international guidelines in everyday practice should be made in order to improve the control rates of hypertension in CEE countries.

High prevalence but low control of hypertension, diabetes and hypercholesterolemia in CEE countries are not only medical problems but also great societal and resource-intensive burden. Coinciding with the transition from communism to the market economy, primary care systems have under-gone re-structuring across the region. Together with improving the health care system, increasing public awareness of these chronic conditions is needed, e.g., by community education, telehealth support and counselling [45].

## Conclusions

In conclusion, in the four CEE study populations, we found high prevalence but low control of hypertension, diabetes, and hypercholesterolemia in both men and women. Awareness rates of hypertension were the lowest in both men and women in the Czech Republic, while awareness rates of hypercholesterolemia were the highest in both men and women in Poland. Polish participants also had the highest rates of awareness, treatment and control of diabetes. The common risk factors for the three chronic conditions were age, gender, education, obesity and alcohol consumption. While the patterns of awareness, treatment and control rates of hypertension, diabetes and hypercholesterolemia somewhat differed between the study populations, the results suggested that efforts should be made in all four countries to control these conditions, including implementation of international guidelines in everyday practice to improve detection and effective management of these conditions.

## Supplementary Information

**Additional file 1.**

## Data Availability

The datasets used and/or analysed during the current study are available from the corresponding author on reasonable request.

## Abbreviations

CEE: Central and Eastern Europe
BMI: Body Mass Index
HAPIEE: Health, Alcohol and Psychosocial factors In Eastern Europe
SBP: Systolic blood pressure
DBP: Diastolic blood pressure
FPG: Fasting plasma glucose
TC: Total cholesterol
CI: Confidence interval
OR: Odds ratio
