# Supplementary material for: Prevalence, awareness, treatment and control of hypertension, diabetes and hypercholesterolemia, and associated risk factors in the Czech Republic, Russia, Poland and Lithuania: a cross-sectional study
Source: BMC Public Health. 2022 May 4;22:883. doi: 10.1186/s12889-022-13260-3 (PMC9066905; doi:10.1186/s12889-022-13260-3)
Supplement: Supplementary file 1 — Additional file 1. [file 12889_2022_13260_MOESM1_ESM.docx]

# **Supplementary materials**

***Table S1 Survey methods in each country***

| Country | Sampling | Blood pressure device | Number of blood pressure measure | Protocol of SBP/DBP calculation | Total cholesterol measurement | Total cholesterol device | Blood glucose measurement | Blood glucose device |
| --- | --- | --- | --- | --- | --- | --- | --- | --- |
| Czech Republic | Multistage stratified random | OMRON M5-I | 3 | Have one blood pressure reading, use it as his/her blood pressure; Have two blood pressure readings, disregard the first one and use the second one as his/her blood pressure; Have three blood pressure readings, disregard the first one and use the mean of the remaining readings as his/her blood pressure. | Measured in a laboratory using plasma samples | Cobas Mira Plus | Measured in a laboratory using plasma samples | Cobas Mira Plus |
| Russia | Multistage stratified random | OMRON M5-I | 3 |  | Measured in a laboratory using blood samples | KoneLab 30i autoanalyser | Measured in a laboratory using plasma samples | KoneLab 30i autoanalyser |
| Poland | Multistage stratified random | OMRON M5-I | 3 |  | Measured in a laboratory using blood samples | Hitachi 917 (Roche) | Measured in a laboratory using plasma samples | Hitachi 917 (Roche) |
| Lithuania | Multistage stratified random | OMRON M5-I | 3 |  | Measured in a laboratory using blood samples | Cobas Mira Plus | Measured by a portable device using whole blood samples^*^ | Glucometer: "Glucotrend" |

*SBP: systolic blood pressure; DBP: diastolic blood pressure; * The whole blood results were converted to plasma readings in Lithuania based on clinical guidance*

***Figure S1 Procedure of sample selection***


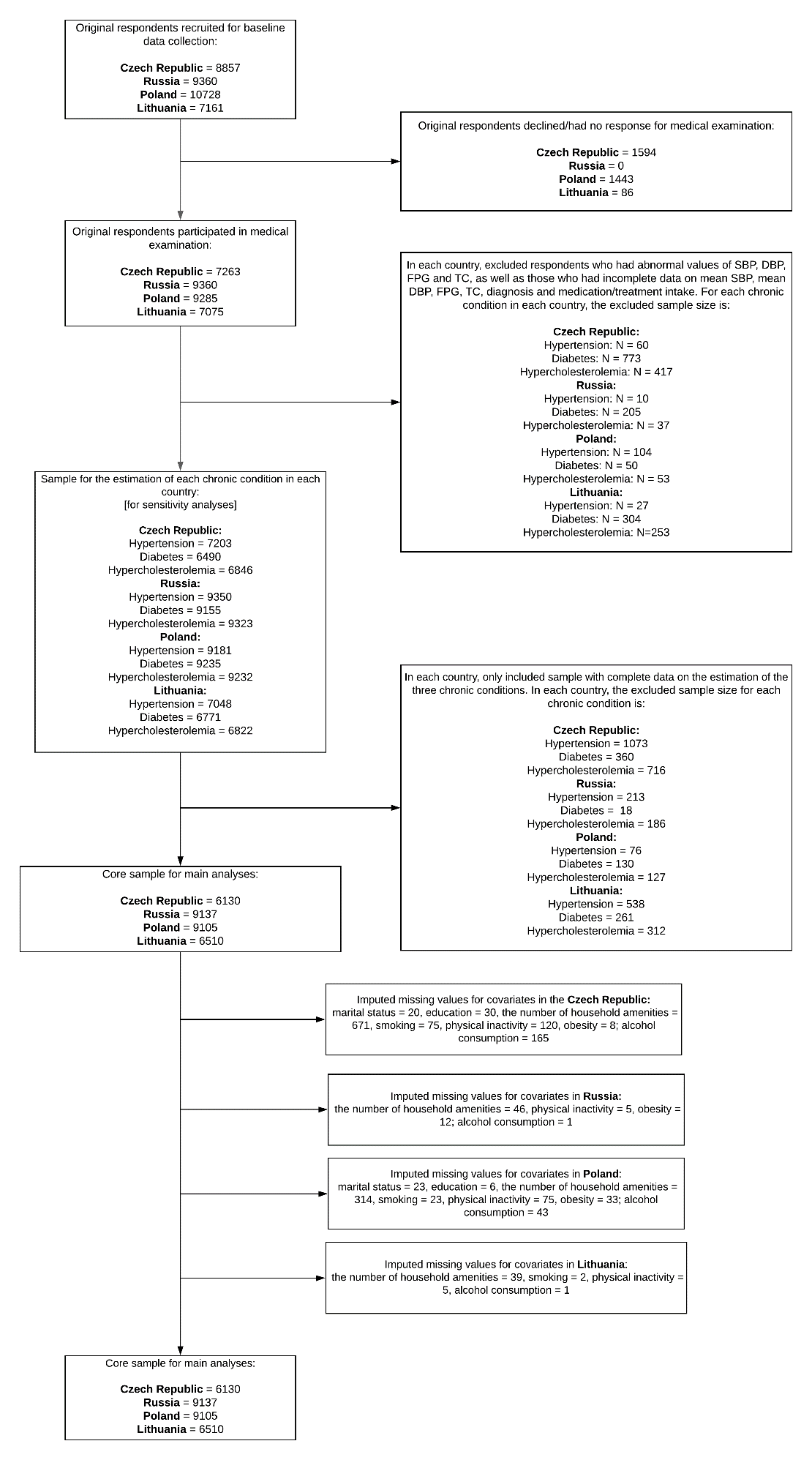


*SBP: systolic blood pressure; DBP: diastolic blood pressure; FPG: fasting plasma glucose; TC: total cholesterol*

***Figure S2 Differences in disease status and risk factors between countries (Czech Republic: reference group)***


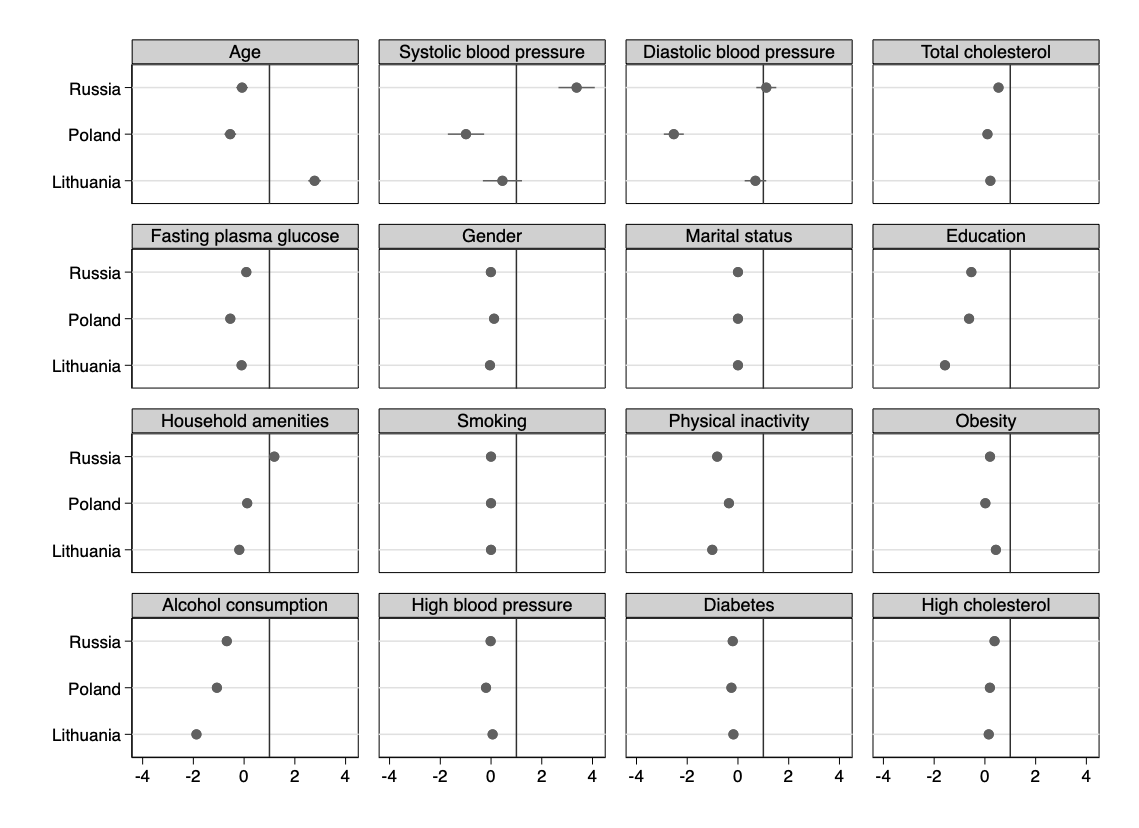


***Table S2 Prevalence, awareness, treatment and control of hypertension, diabetes and hypercholesterolemia in men by country (sensitivity analyses in maximum number of non-missing observations)***

|  | Czech Republic | Russia | Poland | Lithuania |
| --- | --- | --- | --- | --- |
|  | **Proportion (95%CI)** | **Proportion (95%CI)** | **Proportion (95%CI)** | **Proportion (95%CI)** |
| Hypertension | **N=3293** | **N=4259** | **N=4464** | **N=3196** |
|  | **%** | **%** | **%** | **%** |
| Prevalence | 73.03 (71.49–74.52) | 63.35 (61.89–64.78) | 66.71 (65.31–68.08) | 72.68 (71.11–74.20) |
| Awareness | 62.16 (60.20–64.08) | 68.46 (66.68–70.19) | 72.33 (70.69–73.91) | 75.42 (73.63–77.13) |
| Treatment | 50.06 (48.06–52.06) | 34.77 (32.99–36.59) | 55.34 (53.55–57.12) | 48.73 (46.70–50.76) |
| Treatment among awareness | 80.54 (78.45–82.47) | 50.79 (48.50–53.06) | 76.51 (74.67–78.25) | 64.61 (62.34–66.82) |
| Control | 11.27 (10.06–12.60) | 7.45 (6.52–8.50) | 14.94 (13.71–16.27) | 10.85 (9.65–12.18) |
| Control among treatment | 22.51 (20.23–24.96) | 21.43 (18.92–24.17) | 27.00 (24.91–29.20) | 22.26 (19.93–24.78) |
| Diabetes | **N=2939** | **N=4167** | **N=4500** | **N=3050** |
|  | **%** | **%** | **%** | **%** |
| Prevalence | 16.84 (15.53–18.24) | 10.73 (9.82–11.70) | 12.13 (11.21–13.12) | 10.89 (9.83–12.04) |
| Awareness | 54.14 (49.72–58.50) | 30.87 (26.75–35.32) | 77.29 (73.58–80.62) | 54.52 (49.11–59.82) |
| Treatment | 42.22 (37.93–46.63) | 24.61 (20.83–28.83) | 71.98 (68.05–75.59) | 49.70 (44.33–55.08) |
| Treatment among awareness | 77.99 (72.60–82.56) | 79.71 (72.11–85.65) | 93.13 (90.28–95.19) | 91.16 (86.02–94.53) |
| Control | 11.72 (9.16–14.87) | 4.92 (3.26–7.37) | 31.14 (27.38–35.15) | 15.96 (12.39–20.32) |
| Control among treatment | 27.75 (20.07–34.25) | 20.00 (13.49–28.62) | 43.26 (38.42–48.22) | 32.12 (25.40–39.68) |
| Hypercholesterolemia | **N=3161** | **N=4253** | **N=4495** | **N=3075** |
|  | **%** | **%** | **%** | **%** |
| Prevalence | 76.59 (75.08–78.03) | 80.65 (79.43–81.81) | 79.93 (78.74–81.08) | 77.66 (76.15–79.10) |
| Awareness | 36.22 (34.33–38.16) | 11.20 (10.18–12.30) | 46.42 (44.80–48.06) | 22.49 (20.86–24.21) |
| Treatment | 17.68 (16.21–19.25) | 3.79 (3.20–4.48) | 20.79 (19.49–22.15) | 9.42 (8.31–10.66) |
| Treatment among awareness | 48.80 (45.50–52.12) | 33.85 (29.28–38.75) | 44.78 (42.41–47.18) | 41.90 (37.78–46.13) |
| Control | 5.62 (4.77–6.61) | 0.55 (0.35–0.87) | 5.09 (4.42–5.86) | 2.05 (1.55–2.71) |
| Control among treatment | 31.78 (27.52–36.35) | 14.62 (9.48–21.86) | 24.50 (21.54–27.72) | 21.78 (16.84–27.68) |

***Table S3 Prevalence, awareness, treatment and control of hypertension, diabetes and hypercholesterolemia in women by country(sensitivity analyses in maximum number of non-missing observations)***

|  | Czech Republic | Russia | Poland | Lithuania |
| --- | --- | --- | --- | --- |
|  | **Proportion (95%CI)** | **Proportion (95%CI)** | **Proportion (95%CI)** | **Proportion (95%CI)** |
| Hypertension | **N=3910** | **N=5091** | **N=4717** | **N=3852** |
|  | **%** | **%** | **%** | **%** |
| Prevalence | 58.72 (57.17–60.26) | 66.82 (65.52–68.10) | 55.76 (54.33–57.17) | 61.73 (60.19–63.26) |
| Awareness | 69.43 (67.51–71.28) | 85.83 (84.62–86.96) | 81.94 (80.42– 83.36) | 85.37 (83.89–86.73) |
| Treatment | 60.50 (58.48–62.48) | 60.61 (58.96–62.24) | 70.15 (68.37–71.87) | 69.39 (67.50–71.21) |
| Treatment among awareness | 87.14 (85.40–88.70) | 70.62 (68.94–72.24) | 85.61 (84.07–87.03) | 81.28 (79.52–82.92) |
| Control | 21.86 (20.22–23.60) | 15.55 (14.37–16.81) | 26.54 (24.89–28.26) | 22.79 (21.15–24.52) |
| Control among treatment | 36.14 (33.65–38.71) | 25.65 (23.81–27.59) | 37.83 (35.64–40.07) | 32.85 (30.62–35.15) |
| Diabetes | **N=3551** | **N=4988** | **N=4735** | **N=3721** |
|  | **%** | **%** | **%** | **%** |
| Prevalence | 9.60 (8.68–10.62) | 10.61 (9.78–11.49) | 8.34 (7.59–9.16) | 11.29 (10.31–12.34) |
| Awareness | 56.30 (50.97–61.50) | 45.37 (41.16–49.64) | 79.75 (75.48–83.43) | 59.76 (54.98–64.36) |
| Treatment | 39.59 (34.52–44.90) | 38.75 (34.68–42.99) | 75.19 (70.68–79.21) | 51.19 (46.40–55.96) |
| Treatment among awareness | 70.31 (63.43–76.38) | 85.42 (80.34–89.36) | 94.29 (91.10–96.38) | 85.66 (80.73–89.49) |
| Control | 6.45 (4.28–9.62) | 8.13 (6.08–10.79) | 37.72 (33.06–42.62) | 16.19 (12.96–20.04) |
| Control among treatment | 16.30 (10.94–23.59) | 20.98 (15.91–27.13) | 50.17 (44.48–55.85) | 31.63 (25.73–38.18) |
| Hypercholesterolemia | **N=3685** | **N=5070** | **N=4737** | **N=3747** |
|  | **%** | **%** | **%** | **%** |
| Prevalence | 82.61 (81.35–83.80) | 89.72 (88.86–90.53) | 85.75 (84.73–86.72) | 86.04 (84.89–87.12) |
| Awareness | 39.03 (37.31–40.77) | 21.54 (20.37–22.76) | 51.35 (49.82–52.89) | 34.96 (33.33–36.62) |
| Treatment | 16.98 (15.69–18.36) | 4.59 (4.02–5.24) | 24.00 (22.71–25.34) | 12.07 (10.99–13.24) |
| Treatment among awareness | 43.52 (40.72–46.36) | 21.33 (18.87–24.01) | 46.74 (44.61–48.89) | 34.52 (31.79–37.35) |
| Control | 4.20 (3.55–4.98) | 0.35 (0.22–0.57) | 4.01 (3.45–4.66) | 1.55 (1.18–2.04) |
| Control among treatment | 24.76 (21.22–28.67) | 7.66 (4.73–12.16) | 16.72 (14.50–19.20) | 12.85 (9.87–16.57) |

***Table S4 comparing sample characteristics between analytical and excluded sample***

|  | Czech Republic | | | Russia | | | Poland | | | Lithuania | | |
| --- | --- | --- | --- | --- | --- | --- | --- | --- | --- | --- | --- | --- |
|  | **Analytical (N=6130)** | **Excluded (N=1133)** | **P-value** | **Analytical (N=9137)** | **Excluded (N=223)** | **P-value** | **Analytical (N=9105)** | **Excluded (N=180)** | **P-value** | **Analytical (N=6510)** | **Excluded (N=565)** | **P-value** |
| Age  (mean, standard deviation) | 58 (0.09) | 58 (0.21) | 0.189 | 58 (0.07) | 57 (0.48) | 0.253 | 57 (0.07) | 58 (0.53) | 0.296 | 61 (0.09) | 60 (0.33) | 0.004 |
| Gender (%) |  |  |  |  |  |  |  |  |  |  |  |  |
| Men | 45.66 | 46.25 | 0.715 | 45.51 | 47.53 | 0.548 | 48.68 | 47.78 | 0.811 | 44.64 | 54.51 | <0.001 |
| Women | 54.34 | 53.75 |  | 54.49 | 52.47 |  | 51.32 | 52.22 |  | 55.36 | 45.49 |  |
| Marital status (%) |  |  |  |  |  |  |  |  |  |  |  |  |
| Married/Cohobating | 76.51 | 72.50 | 0.015 | 72.30 | 73.09 | 0.531 | 76.93 | 75.00 | 0.829 | 69.49 | 72.39 | 0.356 |
| Single/Divorced/Separated | 14.57 | 17.24 |  | 14.15 | 15.70 |  | 12.72 | 13.89 |  | 16.37 | 14.87 |  |
| Widowed | 8.92 | 10.26 |  | 13.55 | 11.21 |  | 10.35 | 11.11 |  | 14.13 | 12.74 |  |
| Education (%) |  |  |  |  |  |  |  |  |  |  |  |  |
| College education/above | 13.80 | 13.22 | 0.857 | 28.81 | 31.39 | 0.858 | 29.05 | 30.56 | 0.471 | 55.88 | 54.34 | 0.062 |
| Secondary education | 36.92 | 37.71 |  | 34.20 | 33.63 |  | 38.84 | 41.67 |  | 24.58 | 22.12 |  |
| Vocational education | 37.13 | 36.38 |  | 26.53 | 25.11 |  | 20.79 | 20.00 |  | 7.07 | 9.37 |  |
| Primary education/below | 12.15 | 12.69 |  | 10.46 | 9.87 |  | 11.32 | 7.78 |  | 12.47 | 13.81 |  |
| Household amenities (%) |  |  |  |  |  |  |  |  |  |  |  |  |
| High level | 36.27 | 37.32 | 0.604 | 15.41 | 12.22 | 0.296 | 33.16 | 31.03 | 0.180 | 40.67 | 40.57 | 0.699 |
| Middle level | 39.88 | 38.16 |  | 33.05 | 31.67 |  | 40.85 | 36.78 |  | 38.60 | 40.04 |  |
| Low level | 23.85 | 24.53 |  | 51.53 | 56.11 |  | 25.99 | 32.18 |  | 20.72 | 19.39 |  |
| Smoking (%) |  |  |  |  |  |  |  |  |  |  |  |  |
| Non-smokers | 44.51 | 45.39 | 0.824 | 58.21 | 55.61 | 0.562 | 40.78 | 42.22 | 0.679 | 63.46 | 55.42 | <0.001 |
| Ex-smokers | 30.02 | 29.19 |  | 13.67 | 13.00 |  | 29.10 | 26.11 |  | 17.85 | 19.01 |  |
| Current smokers | 25.47 | 25.43 |  | 28.12 | 31.39 |  | 30.11 | 31.67 |  | 18.68 | 25.58 |  |
| Physical inactivity (vigorous activity <2.5 hours/week) (%) |  |  |  |  |  |  |  |  |  |  |  |  |
| No | 13.78 | 17.25 | 0.003 | 6.59 | 6.28 | 0.852 | 10.07 | 9.04 | 0.653 | 5.50 | 6.60 | 0.280 |
| Yes | 86.22 | 82.75 |  | 93.41 | 93.72 |  | 89.93 | 90.96 |  | 94.50 | 93.40 |  |
| Obesity (%) |  |  |  |  |  |  |  |  |  |  |  |  |
| No | 69.55 | 67.85 | 0.255 | 65.11 | 60.18 | 0.129 | 69.17 | 62.64 | 0.065 | 59.66 | 62.08 | 0.264 |
| Yes | 30.45 | 32.15 |  | 34.89 | 39.82 |  | 30.83 | 37.36 |  | 40.34 | 37.92 |  |
| Alcohol consumption (%) |  |  |  |  |  |  |  |  |  |  |  |  |
| None | 11.60 | 13.02 | 0.201 | 15.95 | 13.00 | 0.502 | 33.63 | 34.08 | 0.162 | 47.86 | 45.65 |  |
| <1/month | 26.37 | 24.74 |  | 38.17 | 41.26 |  | 23.88 | 28.49 |  | 26.86 | 25.40 | 0.080 |
| 1–3/month | 20.62 | 22.98 |  | 21.29 | 20.63 |  | 20.04 | 12.85 |  | 19.50 | 20.78 |  |
| 1–4/week | 28.52 | 26.79 |  | 22.03 | 23.77 |  | 18.56 | 20.67 |  | 5.35 | 7.10 |  |
| 5+/week | 12.89 | 12.47 |  | 2.56 | 1.35 |  | 3.88 | 3.91 |  | 0.45 | 1.07 |  |
